# Evaluation of *BLID* and *LOC399959* as candidate genes for high myopia in the Chinese Han population

**Published:** 2010-10-02

**Authors:** Fuxin Zhao, Jian Bai, Wei Chen, Anquan Xue, Chaohua Li, Zhonghui Yan, Hui Chen, Fan Lu, Yongwu Hu, Jia Qu, Changqing Zeng, Xiangtian Zhou

**Affiliations:** 1 School of Optometry and Ophthalmology and Eye Hospital, Wenzhou Medical College, Wenzhou, Zhejiang, China; 2 Beijing Institute of Genomics, Key Laboratory of Genome Sciences and Information, Chinese Academy of Sciences, Beijing, China; 3 State Key Laboratory Cultivation Base and Key Laboratory of Vision Science, Ministry of Health P.R. China and Zhejiang Provincial Key Laboratory of Ophthalmology and Optometry, Wenzhou, Zhejiang, China; 4 Graduate School of the Chinese Academy of Sciences, Beijing, China; 5 Department of Ophthalmology, Eye Hospital of Shenzhen, Shenzhen, Guangdong, China; 6 Department of Ophthalmology, Affiliated Hospital of Nantong University, Nantong, Jiangshu, China

## Abstract

**Purpose:**

BH3-like motif containing, cell death inducer (*BLID*) and *LOC399959* are two genes associated with the single nucleotide polymorphism (SNP) rs577948, which is a susceptibility locus for high myopia in Japanese subjects. The purpose of this study was to determine if *BLID* and *LOC399959* are associated with high myopia in Chinese Han subjects.

**Methods:**

High myopia subjects (n=476) had a spherical refractive error of less than −6.00 D in at least one eye and/or an axial length greater than 26 mm. Genomic DNA was extracted and genotyped from peripheral blood leukocytes of high myopes and controls (n=275). Using a case-control association study of candidate regions, linkage disequilibrium blocks for 19 tag SNPs (tSNPs), including rs577948, harbored within and surrounding the *BLID* and *LOC399959* genes were analyzed on a MassArray platform using iPlex chemistry. Each of the tSNPs had an r^2^>0.8 and minor allele frequency >10% in the Chinese Han population. Haplotype association analysis was performed on Haploview 4.1 using Chi-square (χ^2^) tests.

**Results:**

None of the 19 tSNPs were statistically associated with high myopia.

**Conclusions:**

While rs577948 may be associated with high myopia in Japanese subjects, it and the other tSNPs near the *BLID* and *LOC399959* genes are not susceptibility loci for high myopia in the Chinese Han population. Thus, associations of SNPs with high myopia as determined by Genome-Wide Association Study (GWAS) may be restricted to certain ethnic or genetically distinct populations. Without systematic replication in other populations, the results of GWAS associations should be interpreted with great caution.

## Introduction

Myopia is the most common worldwide ocular disorder. The prevalence of myopia is much higher in Asians than in Caucasians and is an especially important public health issue in Asia. In Western populations, approximately 25% of the decreased vision is caused by myopia, while in some Asian regions, such as China, Singapore, Taiwan, Japan, and Hong Kong, it accounts for 60 – 80% of young adult decreased vision [1]. High myopia is an extreme form of myopia and is usually defined by the presence of an axial eye length greater than 26 mm or a refractive error of less than −6.00 diopters (D). It is usually associated with other ocular disorders such as retinal detachment, macular degeneration, cataract, and glaucoma. For this reason, it is often designated as “pathologic” myopia. High myopia is the fourth most common cause of irreversible blindness [2]. In Asia, the prevalence of high myopia is 1% to 5% [3,4], even reaching 9.1% in some regions [5].

High myopia is a complex disease associated with both genetic and environmental factors. Genetic linkage studies have so far identified 18 chromosomal regions harboring myopia-related genes (MYPs). Of these candidate loci, 11 have been associated with high myopia: MYP1–5 [2,6-9], MYP11 [10], MYP12 [11], MYP13 [12], MYP15 [13], MYP16 [14], and MYP18 [15]. In the same chromosomal regions, many candidate genes associated with high myopia have been studied, such as collagen, type I, alpha 1 (*COL1A1*) [16], transforming growth factor, beta 1 (*TGF beta1*) [17,18], transforming growth factor-beta-induced factor (*TGIF*) [19], lumican (*LUM*) [20,21], hepatocyte growth factor (*HGF*) [22], myocilin (*MYOC*) [23], paired box 6 (*PAX6*) [24,25], and uromodulin-like 1 (*UMODL1*) [26], but none of these are now thought to be responsible for or associated with high myopia.

The genome-wide association study (GWAS) is a powerful research tool, using single nucleotide polymorphisms (SNPs) as markers to identify susceptibility genes of many complex diseases, and it has been successfully applied to identify genetic risk factors for ocular diseases. In 2009, Nakanishi et al. [27] used GWAS to analyze high myopia in a Japanese population. They identified a novel susceptibility locus, rs577948, for pathological myopia at 11q24.1, and within a 200 kb region containing rs577948, they found two genes. BH3-like motif containing, cell death inducer (*BLID*) is a cell death inducer containing a BH3-like motif. It is located approximately 44-kb upstream of rs577948. The other gene, *LOC399959*, is a hypothetical non-coding RNA that encompasses a 114-kb DNA in the region, and rs577948 is located in its second intron. Because of the complexities of associating SNPs with specific disease processes in unique ethnic populations, there is no assurance that the same associations will hold for other ethnic and/or genetically distinct populations. Therefore we genotyped rs577948 and 18 other tag single nucleotide polymorphisms (tSNPs) of the *BLID* and *LOC399959* genes to determine if they were associated with high myopia in a Chinese population.

## Methods

### Subjects

This study was approved by Wenzhou Medical College and by local hospital ethics committees and conducted in accordance with the Declaration of Helsinki principles. All of the subjects for this study were of the Han population from the southern regions of China. They were recruited at the Eye Hospital of Wenzhou Medical College and at local hospitals and informed consent was obtained from each one. The subjects were given complete ophthalmoscopic examinations by local ophthalmologists, including measurements of visual acuity (Topcon RM-8800; Topcon Corp., Tokyo, Japan), axial length (Zeiss IOL Master; Carl Zeiss Meditec, Jena, Germany), spherical refractive error (KOH3 Keratometer; Nikon, Tokyo, Japan), and fundus photography (Canon CR6–45NM Fundus Camera; Canon Inc., Tokyo, Japan).

Individuals with high myopia had a spherical refractive error of less than −6.00 D in at least one eye and/or an axial length greater than 26 mm. Potential subjects with other known ocular or systemic diseases such as Stickler and Marfan’s syndromes were excluded. Controls were recruited and met the following criteria: (1) had no known ocular disease or other genetic diseases or systemic connective tissue disorders associated with myopia, (2) were without family history of high myopia, (3) had spherical refractive error ranging from −0.50 to +2.00 D, and (4) had axial lengths less than 24 mm in both eyes.

### DNA extraction

Genomic DNA was extracted from peripheral blood cells using QIAamp DNA Blood Mini Kit (Qiagen GmbH, Hilden, Germany) according to the manufacturer’s protocol. Briefly, 20 μl Qiagen Protease, 200 μl whole blood, and 200 μl AL Buffer was added to 1.5 ml microcentrifuge tube, respectively. The admixture was blended by pulse-vortexing and incubated at 56 °C for 10 min, then added 200 μl ethanol (96–100%) to mixture, centrifuged at 6,000× g (8,000 rpm) for 1 min, opened the QIAamp spin column and added 500 μl Buffer AW1, centrifuged at 6,000× g (8,000 rpm) for 1 min, added 500 μl Buffer AW2, centrifuged at 20,000× g (14,000 rpm) for 1 min. The isolated DNA was eluted in TE buffer (10 mM Tris-HCl, 0.5 mM EDTA, pH 9.0), and the A_260_/A_280_ optical density was measured by NanoDrop ND1000 (Thermo Fisher Scientific, Waltham, MA). It was then stored at −80 °C before use.

### SNP selection and genotyping

To determine the association between the SNPs and the high myopic subjects, we used the tag single nucleotide polymorphisms (tSNPs) approach. The tSNPs of the *BLID* and *LOC399959* genes were selected from the public Single Nucleotide Polymorphism database build 126 and the phase data of the HapMap Project release 27. Each tSNP met the following criteria: r^2^>0.8 and minor allele frequency (MAF)>10% in the Chinese Han population [28,29]. We selected 19 tSNPs to tag the linkage disequilibrium (LD) blocks harbored within and surrounding the *BLID* and *LOC399959* genes (Table 1).

**Table 1:** tSNP Genotyping of the *BLID *and *LOC399959 *genes in high myopia and control subjects.

tagSNPs	Major allele	Freq of case	Freq of control	p value	OR (95% CI)	P_permutation_*
rs512932	G	0.2500	0.2400	0.6209	0.93 (0.71-1.22)	1.0000
rs531897	C	0.3365	0.3297	0.9517	0.99 (0.77-1.26)	1.0000
rs547008	T	0.2400	0.2365	0.6875	0.94 (0.72-1.23)	1.0000
rs577948	A	0.4801	0.5054	0.5813	0.93 (0.74-1.17)	1.0000
rs638742	C	0.4329	0.4314	0.4008	1.10 (0.87-1.39)	0.9978
rs657514	C	0.4316	0.4388	0.5696	1.06 (0.85-1.34)	1.0000
rs664409	T	0.3029	0.3455	0.1797	0.84 (0.65-1.08)	0.9058
rs683185	A	0.2787	0.2996	0.4131	0.89 (0.69-1.16)	0.9984
rs947893	C	0.3222	0.3321	0.5805	0.93 (0.72-1.19)	1.0000
rs1143770	C	0.4609	0.4532	0.6015	0.94 (0.75-1.18)	1.0000
rs1615327	G	0.4948	0.4820	0.8354	1.02 (0.81-1.29)	1.0000
rs1816158	C	0.3936	0.3778	0.9546	0.99 (0.78-1.25)	1.0000
rs1971734	G	0.2808	0.2392	0.2182	1.18 (0.90-1.53)	0.9467
rs2241490	T	0.3424	0.3058	0.1217	1.22 (0.94-1.58)	0.7896
rs6589913	T	0.2364	0.2284	0.4109	1.12 (0.85-1.48)	0.9983
rs7119477	C	0.3122	0.3370	0.1328	0.81 (0.63-1.06)	0.8183
rs10790486	G	0.4102	0.4152	0.3439	0.88 (0.69-1.13)	0.9935
rs11604461	C	0.2146	0.1715	0.0726	1.30 (0.97-1.74)	0.6041
rs12273515	G	0.4292	0.4478	0.9673	1.00 (0.79-1.26)	1.0000

The asterisk indicates that 50,000 permutations were performed. In the Table, OR indicates odds ratio and CI indicates confidence interval.

DNA samples were initially diluted to ~10 ng/µl as measured with PicoGreen Dye (Invitrogen, Carlsbad, CA) in a NanoDrop ND3300 fluorospectrometer (Thermo Fisher Scientific, Waltham, MA). The tSNPs of the *BLID* and *LOC399959* genes were genotyped on MassArray platform using iPlex chemistry (Sequenom Inc., San Diego, CA) according to the manufacturer’s instruction. Loci with a call rate >0.8 were selected for association analysis on PLINK 1.07 and haplotype analysis on Haploview 4.1 [30].

For clinical data, means and standard errors of the means were determined. We evaluated the allele frequencies of sequence alterations in patients and controls using χ^2^ tests in a logistic regression model where age and gender were included as covariates. Allelic frequencies of detected SNPs in controls were also assessed for Hardy–Weinberg equilibrium. We also performed haplotype association analysis on Haploview 4.1 using χ^2^ tests. P-values of both single SNPs and haplotypes were corrected using permutation test after running 50,000 times.

## Results

### Clinical data

Bilateral high myopia was present in 476 subjects (177 men, 299 women) with a mean age of 36.8±0.7 years (range: 5–77 years), and the age of onset was 14.3±0.5 years. There were 275 control subjects (152 men; 123 women) with an age of 22.3±0.3 years (range: 17–59 years). For subjects with high myopia, the spherical refractive errors of the right and left eyes were −15.05±-0.28 D and −14.57±-0.31 D, respectively. The axial lengths of the right and left eyes were 29.83±0.12 mm and 29.55±0.14 mm, respectively.

### Association analysis

After tSNP analysis of the *BLID* and *LOC399959* genes, none of the nineteen tested tSNPs demonstrated significant association with high myopia (Table 1). Eighteen, including rs577948, failed to reach a nominal significant level (p=0.05). One tSNP, rs11604461, was nearly significant (p=0.07, Table 1), but the permutation test (Ppermutation=0.6041, Table 1) showed it was not a true association. Thus, the results of genotyping indicated that there were no significant differences in the tSNPs between high myopia and controls in the tested region.

**Figure 1:**
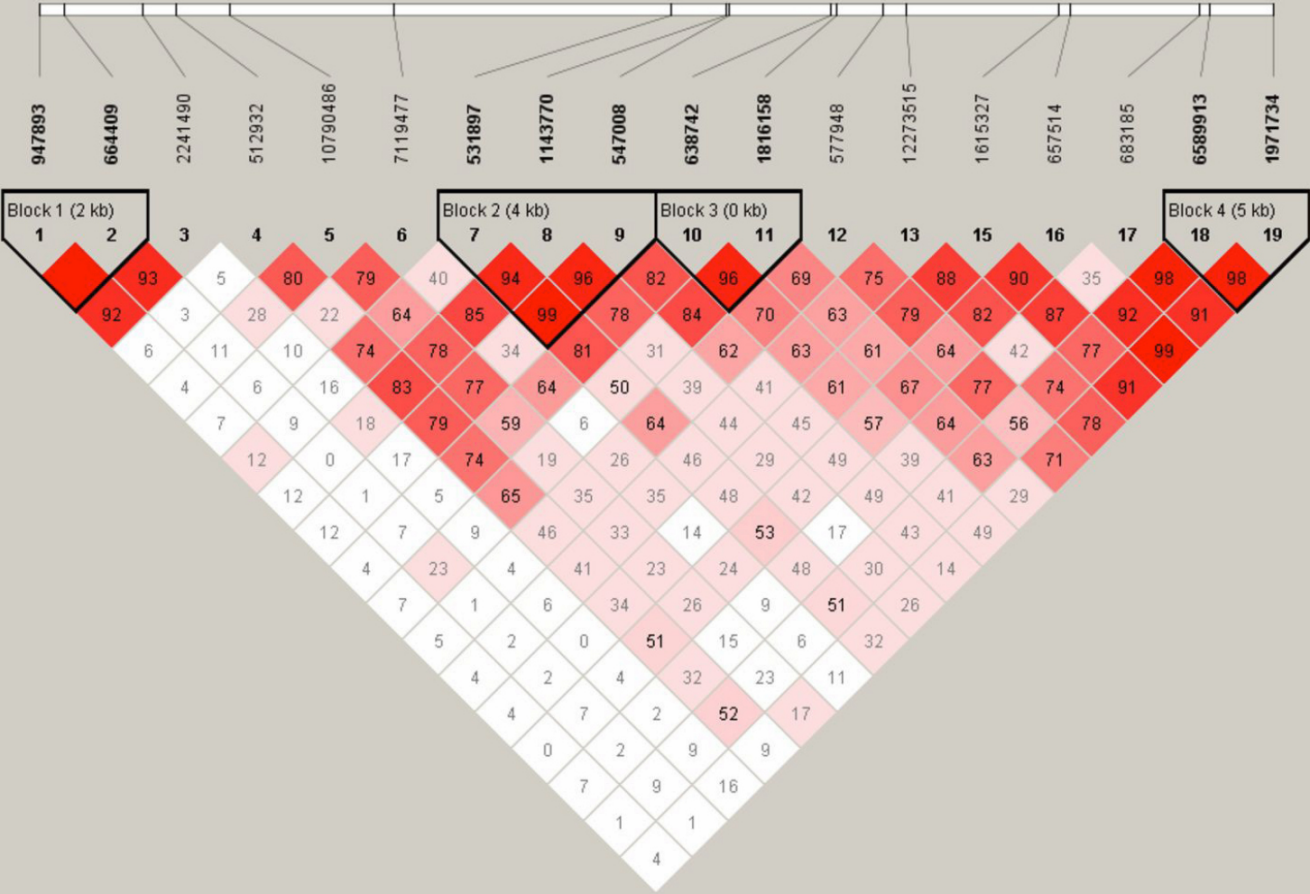
The LD patterns of the *BLID* and *LOC399959* cluster region on 11q24.1 in Chinese Han populations of high myopia.

Haplotype analysis showed that there were five LD blocks in the *BLID* and *LOC399959* cluster regions (Figure 1). After removing haplotypes with frequencies less than 5%, we analyzed haplotype associations on the five blocks. After 50,000 permutations, there were no significant differences between the high myopia and control groups (Table 2).

**Table 2:** Haplotype analysis of the *BLID *and *LOC399959 *genes in high myopia and control subjects.

**Block**	**Haplotype**	**Freq**	**Freq of case**	**Freq of control**	**χ^2^**	p value*	P_permutation_**
**Block 1**							
	TC	0.36	0.374	0.322	4.143	0.0418	0.5573
	CC	0.33	0.323	0.332	0.148	0.7004	1.0000
	TT	0.32	0.303	0.346	2.902	0.0885	0.8196
**Block 2**							
	TTC	0.53	0.529	0.537	0.078	0.7793	1.0000
	CCT	0.24	0.238	0.229	0.144	0.7041	1.0000
	TCC	0.13	0.134	0.13	0.06	0.806	1.0000
	CCC	0.09	0.089	0.094	0.105	0.7465	1.0000
**Block 3**							
	CT	0.43	0.428	0.424	0.023	0.8806	1.0000
	GC	0.38	0.391	0.372	0.529	0.4672	1.0000
	GT	0.18	0.176	0.195	0.841	0.3591	0.9996
**Block 4**							
	GT	0.44	0.429	0.448	0.499	0.4798	1.0000
	CT	0.37	0.356	0.381	0.952	0.3291	0.9988
	CC	0.20	0.215	0.171	4.229	0.0397	0.5406
**Block 5**							
	GC	0.5	0.483	0.535	3.703	0.0543	0.6453
	GG	0.26	0.28	0.237	3.392	0.0655	0.7201
	TC	0.23	0.236	0.226	0.181	0.6709	1.0000

The asterisk indicates that differences in the estimated haplotype frequency were examined by the χ^2^ test. The double asterisk indicates that 50,000 permutations were performed.

## Discussion

GWAS has been used to successfully identify susceptibility SNPs and genes in many complex disorders, such as diabetes mellitus [31], obesity [32], prostatic cancer [33], and aged-related macular degeneration [34-36]. However, a key question of GWAS is whether or not the results can be replicated in subjects of different ethnicities. The aim of this study was to determine if the *BLID* and *LOC399959* genes are disease susceptibility loci for high myopia in the Chinese Han population. The SNP rs577948 was the first to be validated by GWAS as a high myopia susceptibility locus in Japanese subjects [27]. However in the Chinese Han population, we found no significant association with high myopic patients and controls by genotyping 19 tSNPs, including rs577948, of the *BLID* and *LOC399959* genes.

This study can be seen as the second stage of a traditional GWAS analysis. Therefore we used the GWApower [37], a R package for assessing the power of genome-wide association studies, as the simulation tool to compare the detection power of our sample size with the previous study in a Japanese population. With the p-value threshold of 10^−6^, relative risk of 1.5, and MAF of 0.4 according to their first stage results [37], the power of Japanese second stage study with 533 cases and 977 controls is 0.132. That is much higher than ours at 0.018. However, the criteria for the control subjects in our study are more stringent, and our health controls enabled us to get a higher relative risk value in the current sample size. Using 1.7 as the relative risk, the power of our study reached 0.114, which means that our study has the same power to detect the association signal in *LOC399959* as in the Japanese study.

In addition, by both χ^2^ test and logistic regression, the mean ages were significantly different between our samples and the Japanese study for both cases and controls. However, this should not impact our conclusion because high myopia is an early onset disease, usually occurring no later than the puberty years. In neither study was gender distribution completely matched for case and control groups. This should also not influence the conclusions as gender is not correlated with the occurrence of high myopia.

There may be two reasons that our results do not replicate those of the Japanese study. First, high myopia is a complex disease that is associated with high genetic heterogeneity. With studies that use different recruitment criteria, the genetic background of the patients selected for analysis could differ significantly from one another. Second, study populations composed of different ethnicities will have different genetic backgrounds. For these populations, the allelic frequencies of SNPs are likely to be different.

Thus far, the results are very limited for replication studies that have attempted to verify myopia susceptibility genes identified in association studies. This is not a surprise since it is common in complex diseases that the significant association signals in one investigation appear to be negative in other analyses. There may be several reasons for such phenomena, including different genetic backgrounds between populations and minor diversity in sample collection, clinical measurement, and statistical analysis. Considering the relatively minor contribution of each genetic factor to a complex disease in comparison with Mendelian inheritance, trivial diversity may result in differences in significance for an association signal. Furthermore, various ethnic groups may have different susceptibility genes in one disease-related pathway. Therefore, although our findings did not confirm that the *BLID* and *LOC399959* genes play a genetic role in Chinese patients with high myopia, we cannot rule out the possibility that other susceptibility genes in related molecular pathways may be involved. Also, for any associated candidate gene, perhaps meta-analysis, i.e., the combination of several high myopia association studies, will be the ultimate method for determination of a true or false positive signal, as shown by recent association studies on diabetes and a few other complex diseases [38-43]. To reach this goal, certainly more independent association studies in various ethnicities are needed.
